# Supplementary material for: Synergistic AML Cell Death Induction by Marine Cytotoxin (+)-1(R), 6(S), 1’(R), 6’(S), 11(R), 17(S)-Fistularin-3 and Bcl-2 Inhibitor Venetoclax
Source: Mar Drugs. 2018 Dec 19;16(12):518. doi: 10.3390/md16120518 (PMC6316187; doi:10.3390/md16120518)
Supplement: Supplementary file 1 [file marinedrugs-16-00518-s001.pdf]

SUPPLEMENTARY MATERIAL

Synergistic AML cell death induction by marine cytotoxin (+)-1(*R*), 6(*S*), 1′(*R*), 6′(*S*), 11(*R*), 17(*S*)-fistularin-3 and Bcl-2 inhibitor Venetoclax

Cristina Florean et al.

Supplementary Figure 1

11-*epi*-Fistularin-3 (11R,17S)

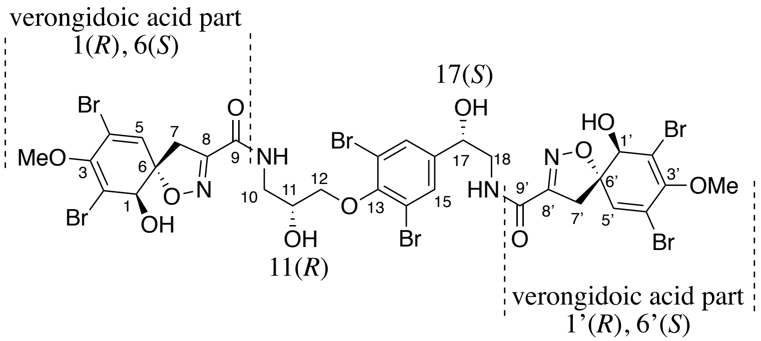

(+)-1(*R*), 6(*S*), 1′(*R*), 6′(*S*), 11(*R*), 17(*S*)-Fistularin-3 (1)
(*described in this work*)

(A) ^{1}H NMR spectrum of (+)-11R, 17S-fistularin-3 in Acetone-d6, 500MHz, 298K.

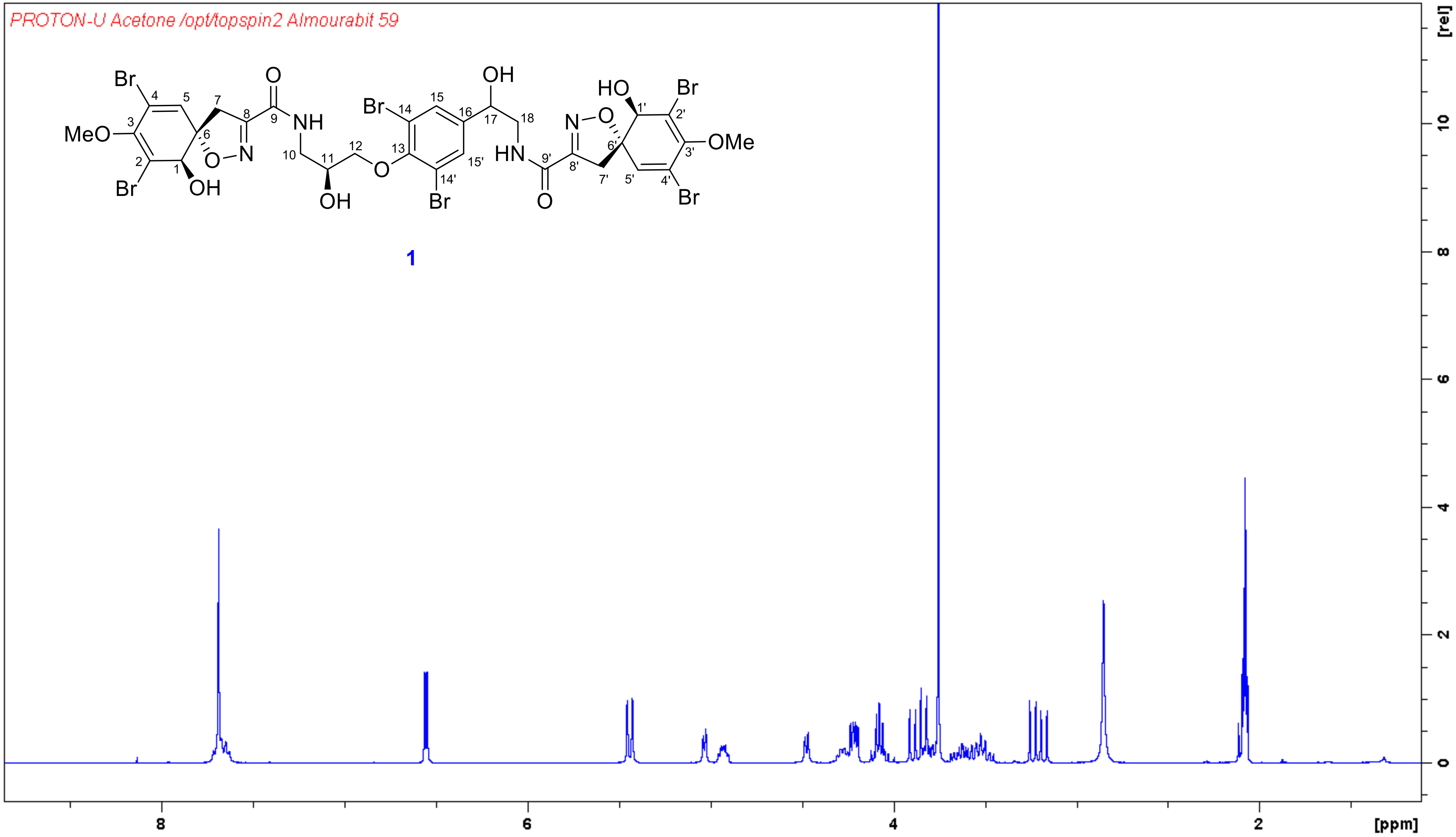

(B) ^{13}C NMR spectrum of Fistularin -3 in Acetone-d6, 125MHz, 298K.

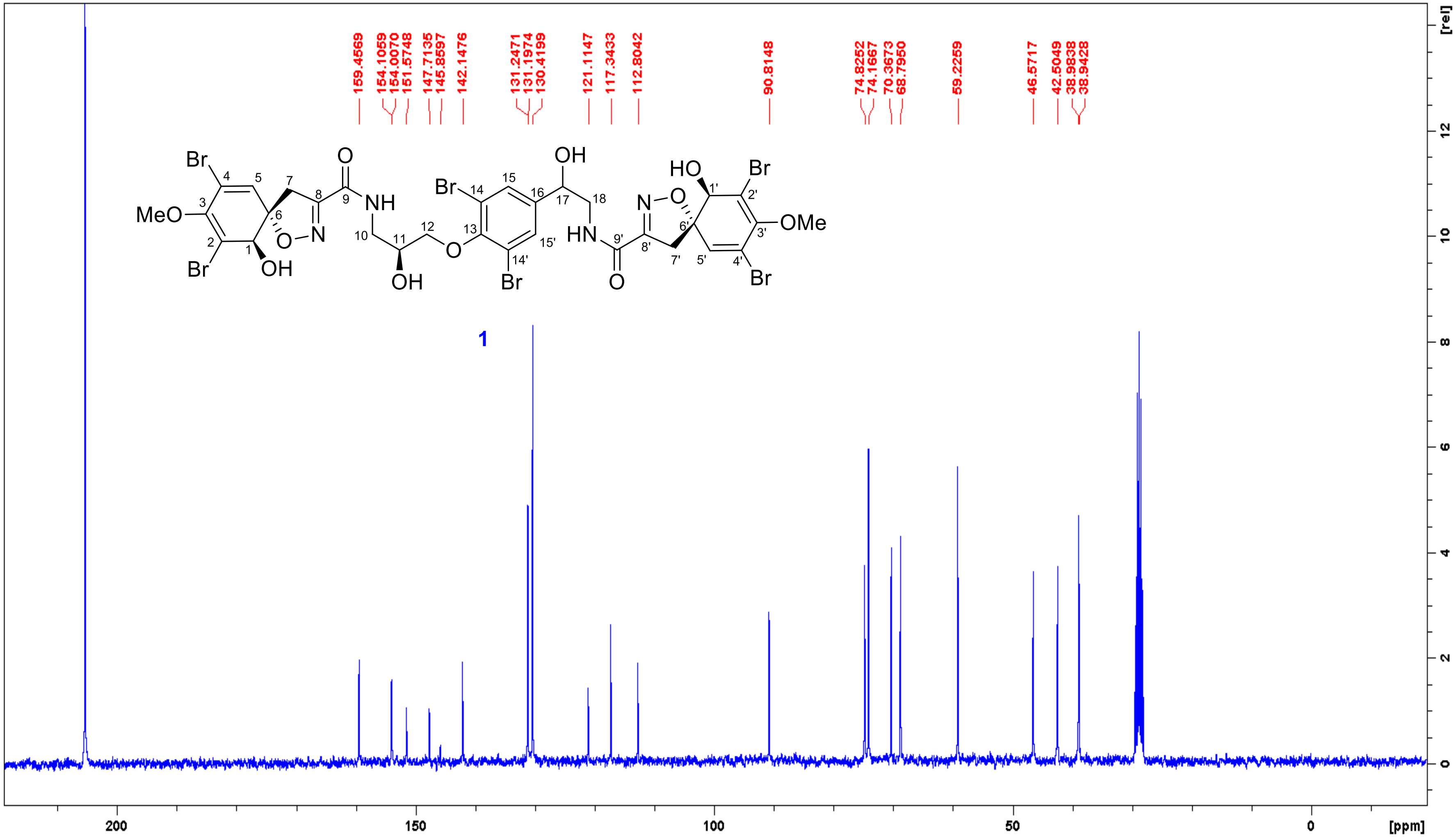

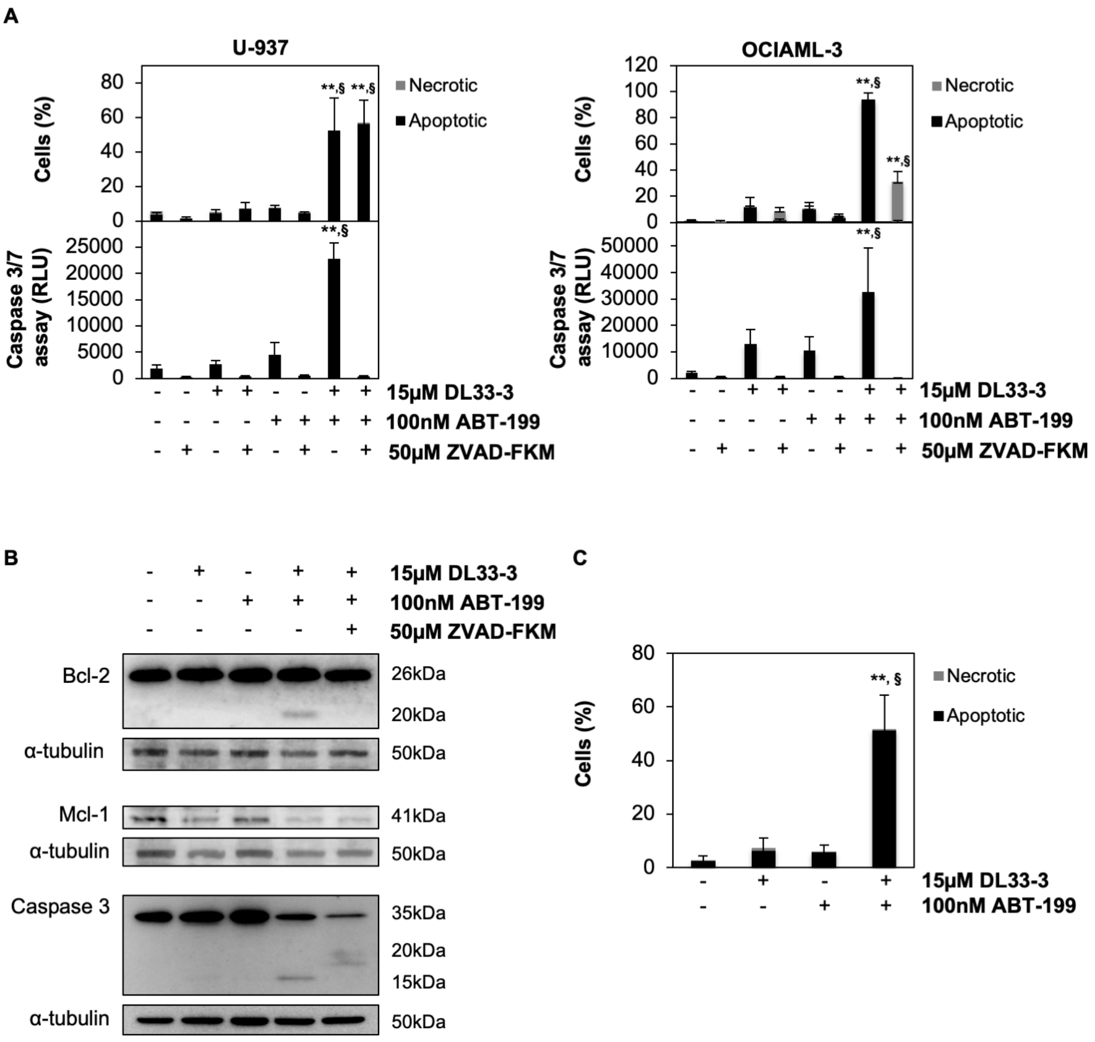

Supplementary Figure 2. Effect of combination treatments of RS-F3 and ABT-199 in AML cell lines in presence of zVAD-FMK and concomitant treatments. (A) Nuclear morphology analyses (upper panel) and caspase 3/7 activity assay (lower panel) in U-937 and OCIAML-3 cells treated with RS-F3 (24h) and then ABT-199 (16h) in presence or absence of 50µM zVAD-FMK. (B) Western blotting analyses in U-937 cells treated as described above. Blots are representative of three independent experiments. (C) Nuclear morphology analysis of U-937 cells treated concomitantly with RS-F3 and ABT-199 for 18h. All histograms represent the mean ± SD of three independent experiments. Asterisks indicate statistical difference respect to control. The symbol § indicates statistical difference of combination treatments respect to both compounds taken alone. * p≤ 0.05, ** p≤ 0.01; §≤ 0.01. RLU: relative luminescence units.

A

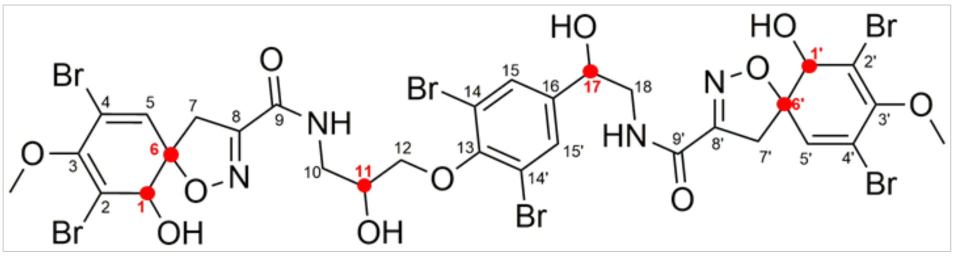

B

Stereo-isomers	Global Energy	Stereo-isomers	Global Energy	Stereo-isomers	Global Energy	Stereo-isomers	Global Energy
RS**RS**SR	-48.22	SR**RS**RR	-38.89	RRSSRS	-35.91	SSSRRR	-32.87
SS**RS**RR	-45.15	RRRRSS	-38.69	SRRRRR	-35.90	RSSRRR	-32.38
RRSSRR	-44.86	RRSRSR	-38.65	RR**RS**RS	-35.50	RSRRRS	-32.31
SRSSRR	-44.84	SRRRSR	-38.41	SSSSSS	-35.28	SSSRRS	-31.89
RS**RS**RS	-44.34	RS**RS**RR	-38.38	SR**RS**RS	-35.18	RSSSSS	-31.42
RS**RS**SS	-42.91	SRSSSS	-37.94	RSSRSR	-35.14	SRSSSR	-31.29
SS**RS**RS	-42.41	SSSSRS	-37.80	SR**RS**SS	-35.06	RSSRRS	-30.81
SSSRSR	-42.33	RRRRSR	-37.78	RSSRSS	-35.04	RR**RS**SR	-30.72
RRSRRS	-41.07	SRSRRR	-37.45	SSRRRR	-35.00	SSRRSS	-29.84
RR**RS**RR	-41.05	SRSRRS	-37.45	RSRRRR	-34.70	SSRRSR	-29.18
SRSSRS	-40.74	SRRRRS	-37.02	RSRRSS	-34.66	SSSRSS	-27.92
SS**RS**SR	-39.88	RRSSSS	-36.91	RRSSSR	-34.36	RRRRRS	-27.61
SS**RS**SS	-39.69	SSSSSR	-36.87	SRRRSS	-34.34	RSSSSR	-27.58
RRSRRR	-39.38	RSSSRS	-36.58	RRSRSS	-34.02	RRRRRR	-27.09
RSRRSR	-39.18	SSRRRS	-36.42	SSSSRR	-33.39	RSSSRR	-25.67
SRSRSR	-38.92	SR**RS**SR	-36.21	RR**RS**SS	-32.96	SRSRSS	-25.04

Supplementary Figure 3. (A) The configuration of fistularin-3. Positions of six chiral centers of fistularin-3 were marked with red dots and carbon numbers are labeled. **(B) Global energy scores for all 64 stereoisomers of fistularin-3.** C11(R), C17(S) stereoisomers are emphasized as bold text in red. Four C11-C17 diastereoisomers with established C1(R), C6(S), C1′(R), C6′(S) configuration are shown in blue boxes.
